# Considering the concept of nutritional modification coupled with phytase supplementation for reducing *Campylobacter jejuni* in broilers

**DOI:** 10.3389/fvets.2025.1686545

**Published:** 2025-10-31

**Authors:** Kirsty Gibbs, Abiodun Bello, Sasha van der Klein, Charlotte Poulsen, Karsten Kragh, Leon Marchal

**Affiliations:** ^1^Danisco Animal Nutrition & Health (IFF), Oegstgeest, Netherlands; ^2^Danisco Nutrition and Biosciences (IFF), Brabrand, Denmark

**Keywords:** broiler, *Campylobacter*, food safety, Iron, nutritional virulence, phosphorus, phytase

## Abstract

*Campylobacter jejuni* remains a significant health concern in humans, with the consumption of contaminated poultry meat being the primary source. Nutritional virulence could be utilized to help reduce *Campylobacter* in poultry, specifically by reducing dietary iron (Fe) and phosphorus (P), which appear to be essential for *Campylobacter* growth and persistence, in combination with phytase supplementation to meet bird mineral requirements. We discuss the scientific basis of this hypothesis and present results of a small-scale proof-of-concept broiler study comprising: (1) control: mixed grain commercial diet; (2) added Fe- and inorganic phosphate (iP)-free premix: as control but formulated without Fe in the mineral premix and without added iP, with increased phytase [phytase unit (FTU)/kg per phase] and higher associated matrix values for Ca, metabolizable energy (ME), and digestible amino acids (AA) vs. control. Over the 42-day (d) trial, birds exhibited similar (non-significantly different) livability and growth performance. Average cecal *Campylobacter* loads were numerically 7.7-fold lower (87% reduction) in the added Fe- and iP-free treatment relative to the control [4.90 × 10^7^ colony forming units (CFU)/g vs. 3.78 × 10^8^ CFU/g, respectively; *p*-value = 0.12]. In addition, the range in recorded loads of *Campylobacter* was wide in both treatments, but the upper end of the range was 1 log10 units lower in added Fe- and iP-free vs. control (2.97 × 109 vs. 2.45 × 1,010), which suggests a reduced upper limit of colonization and reduction in average *Campylobacter* levels. Although caution is warranted on the numerical results, we believe they should encourage further ideations, investigations, and larger scale applications in the future.

## 1 Introduction

Campylobacteriosis, caused predominantly by the gram-negative bacterium *C. jejuni*, is the most reported gastrointestinal disease among humans in the EU: ~46.9 cases per 100,000 population were reported in 2022 (1). It is primarily a foodborne zoonotic disease acquired from the consumption of infected meat, especially poultry meat. *Campylobacter* is generally found in the avian gastrointestinal tract, residing predominantly and at high levels in the blind-ended caeca [Beery et al., 1988; (2)]. However, it can become pathogenic under certain situations, for example, among susceptible host populations (young or immunocompromised birds) under conditions of environmental stress or when birds are infected with certain *Campylobacter* species that are always pathogenic. Numerous on-farm postharvest and processing control strategies have been developed, including biosecurity measures, feed and water additives, bacteriocins, bacteriophage therapy, vaccination, and processing interventions (3), but they are not optimally effective, and flock colonization rates often remain high. New *Campylobacter* control approaches are continuously being sought, and those that build upon existing measures used for other purposes could be advantageous from a cost standpoint. This perspective article considers the concept of utilizing nutritional modifications as a potential approach to reduce *C. jejuni* in broilers.

## 2 Nutritional modification as a potential tool for reducing *Campylobacter*

Nutritional virulence is the exploitation of host nutritional resources by pathogens to support their existence. As research continues to bridge the gap between nutrition and health, focus on nutritional virulence is growing. To date, research has demonstrated that certain dietary nutrients, often oversupplied in commercial diets, promote *Campylobacter* survival and persistence in the poultry digestive tract. Specifically, it has been shown that Fe is essential for *Campylobacter* colonization, growth, and virulence. Ferrous (Fe^2+^) and ferric (Fe^3+^) Fe stimulate biofilm formation, which contributes to the persistence and survival of *C. jejuni* under stress conditions (4). Miller et al. (5) characterized the cellular uptake and processing mechanisms through which host Fe sources are sequestered and utilized by *C. jejuni* for survival and proliferation. These Fe sources include both direct sources, such as host heme compounds and transferrin, from which Fe is extracted by *C. jejuni* via contact-dependent or surface receptor binding process (6), as well as indirect sources, involving the sequestration of Fe from siderophores secreted by other bacteria residing in the gut microbiome (7). It is plausible that precise control of the Fe content in the diet to the required dose could reduce *Campylobacter* growth and persistence by reducing access to free Fe. Furthermore, P is another nutrient critical for *Campylobacter* growth and colonization. It is utilized for intestinal adhesion as part of the high-affinity phosphate transporter PstSCAB, which has been identified as a target for potential *C. jejuni* control methods (8). It is supplemented in conventional broiler diets in the form of iP, often at levels far higher than bird requirements, according to NRC recommendations (9). This raises the question as to whether better optimization of diets for P availability could also enable *Campylobacter* control.

## 3 Supplemental phytase

More precise diet formulation, in which diets are optimized to meet but not exceed bird nutritional requirements, is a major area of current research among poultry nutritionists. This involves careful consideration of how diets are formulated (for example, the levels of total, available, or digestible nutrients) and the ingredients and feed additive biotechnologies to be included in the diet. Such research could be linked to the concept of reducing nutritional virulence. For example, microbially derived phytase is added routinely to broiler diets, primarily to increase the bioavailability of P, which is otherwise primarily bound within plant-based ingredients in the form of phytate (IP_6_, hexakisphosphate). The biochemical properties and activity for hydrolyzing phytate varies from one type to another (10), but its efficacy in broilers for increasing the digestibility of P and certain other nutrients is well documented [recently reviewed by (11)]. This efficacy enables the digestible P content of the diet [and that of other “released” nutrients, including calcium (Ca), AA, energy, and sodium (Na)] to be reduced to prescribed amounts (known as “matrix values”) and accounts for the expected contribution of the enzyme. If added iP could be totally removed from the diet and replaced by phytase, as demonstrated in a proof-of-concept study involving a phytase produced by *Trichoderma reesei* [PhyG, (12)], it might help reduce *Campylobacter* colonization, due to the bacteria’s dependence on P for successful colonization. Furthermore, phytate has a strong affinity for trace minerals at intestinal pH, forming difficult-to-digest complexes (13). This reduces the bioavailability of trace minerals in feed raw materials, even when present in quantities that would otherwise meet bird requirements. A fast-acting phytase with high activity at low pH can prevent trace mineral-phytate complex formation, thus reducing the need to supplement trace minerals in the diet (14). Hence, it is hypothesized that the reduction or removal of added Fe from an all-vegetable diet supplemented with phytase may also help reduce *Campylobacter* levels.

## 4 Early research findings

A small-scale research study conducted by the present authors to test the hypothesis that dietary removal of added Fe and iP, alongside appropriate phytase supplementation and matrix application to an all-vegetable diet, could lower cecal *C. jejuni* colonization without affecting the growth of broilers over 42 d. A completely randomized design with two treatments and three dietary phases (1 to 14, 15 to 28, and 29 to 42 ds of age) was employed. Treatments comprised: (1) commercial control: corn-wheat-soybean meal-based diet with phytase supplemented at 1,000 FTU/kg during all phases and with associated matrix values applied for available P, Ca, digestible AA, and ME; (2) added Fe- and iP-free: formulated without Fe in the mineral premix and without added iP, with phytase added at 3,000, 2,000, and 2,000 FTU/kg per phase, and associated higher matrix values applied for Ca, ME, and digestible AA. Diets were fed ad libitum to 500 Ross 308 male broilers housed in 20 floor pens with 25 birds/pen and 10 pens/treatment. The phytase was a bacterial 6-phytase variant produced in *T. reesei*, supplied by Danisco Animal Nutrition & Health (IFF, Oegstgeest, Netherlands). The calculated and analyzed nutrient composition of the diet ingredients has been listed in Table 1. Birds were batch-tested for *Campylobacter* negativity prior to randomization, and all birds were exposed to the bacteria through oral challenge with ~1.0 × 10^6^ CFU/mL (~1.0 × 10^5^ CFU/bird) of *C. jejuni* (JB strain, isolated from poultry) on d 14. Levels of *C. jejuni* in the ceca of birds at 42 ds of age (CFU/g) were determined by culture-dependent methods. All data were analyzed using JMP 16 software. Performance data (available upon request) were analyzed using one-way ANOVA with mean value segregation using Tukey’s Honest Significant Difference (HSD) test. The significance was defined as *p*-value < 0.05. Due to the nonparametric nature of microbial data, the Wilcoxon rank-sum test was used (Table 2).

**Table 1:** Ingredients and calculated nutrient composition of the treatment diets, % as fed, unless otherwise stated.

Ingredients	Dietary phases					
	Starter (1 to 14 ds of age)		Grower (15 to 28 ds of age)		Finisher (29 to 42 ds of age)	
	Control	Added Fe- and iP-free	Control	Added Fe- and iP-free	Control	Added Fe- and iP-free
Corn	33.84	38.90	39.89	44.97	44.19	48.09
Wheat	10.00	10.00	10.00	10.00	10.00	10.00
Soybean meal	34.59	34.58	30.49	29.15	26.73	26.24
Rapeseed meal	5.00	5.00	5.00	5.00	5.00	5.00
Sunflower meal	2.26	2.18	1.83	2.01	2.56	2.56
Maize gluten meal	5.66	2.94	4.35	2.52	3.40	1.58
Soy oil	4.87	3.59	5.10	3.80	5.26	4.31
Limestone	0.27	1.46	0.52	1.24	0.64	1.01
Dicalcium phosphate	1.91	–	1.26	–	0.77	–
L-lysine HCL	0.34	0.29	0.29	0.27	0.27	0.24
DL-methionine	0.31	0.29	0.28	0.26	0.26	0.25
L-threonine	0.13	0.11	0.11	0.09	0.09	0.08
Mineral premix—with Fe^1^	0.17	–	0.12	–	0.12	–
Mineral premix—without Fe^2^	–	0.17	–	0.12	–	0.12
Vitamin premix^3^	0.05	0.05	0.05	0.05	0.05	0.05
Coccidiostat (amprolium)	0.01	0.01	0.05	0.05	0.05	0.05
Sodium bicarbonate	0.44	0.25	0.42	0.24	0.41	0.23
Sodium chloride	0.18	0.19	0.20	0.19	0.20	0.20
Phytase (PhyG), FTU/kg	1,000	3,000	1,000	2,000	1,000	2,000
Calculated nutrients						
Metabolizable energy, kcal/kg	2,945	2,896	3,020	2,977	3,070	3,036
Crude fat	7.93	6.75	8.21	7.03	8.48	7.61
Starch	28.84	31.50	32.33	35.15	34.61	36.71
Calcium	0.80	0.71	0.70	0.61	0.60	0.52
Available phosphorus	0.35	0.14	0.27	0.13	0.21	0.12
Total P	0.81	0.42	0.65	0.40	0.55	0.39
Crude protein	25.07	23.71	22.75	21.44	20.98	19.91
Digestible Lys	1.32	1.27	1.18	1.13	1.08	1.04
Digestible Met + Cys	1.00	0.94	0.92	0.86	0.86	0.81
Digestible Thr	0.88	0.83	0.79	0.74	0.72	0.68
Digestible Trp	0.23	0.23	0.21	0.20	0.19	0.19
Digestible Arg	1.40	1.37	1.27	1.22	1.17	1.14
Digestible Val	1.00	0.95	0.91	0.86	0.84	0.80
Phytate phosphorus	0.31	0.31	0.30	0.30	0.29	0.29
Analyzed constituents						
Iron, mg/kg	208	134	163	106	146	80
Phytase, FTU/kg	979	3,219	1,243	2,049	907	2,053

Calculated based on CVB feed Table 2021 (20).

1 Supplied per kilogram of diet: K Manganese (Mn), 120 mg; Zinc (Zn), 120 mg; Iron (Fe), 20 mg; Copper (Cu), 16 mg; Selenium (Se), 0.3 mg; and Iodine (I), 1.25 mg.

2 Supplied per kilogram of diet (0.17% in starter diets at 0.12% in grower and finisher diets): 0.1% of the premix equates to Manganese (Mn), 120 mg; Zinc (Zn), 120 mg; Iron (Fe), 0 mg; Copper (Cu), 16 mg; Selenium (Se), 0.3 mg; and Iodine (I), 1.25 mg.

3 Vitamin premix per kilogram of finished feed: vitamin A, 3,750 IU; vitamin D3, 2000 IU; vitamin E, 16 IU; vitamin B12 (cobalamin), 10 μg; biotin, 0.08 mg; menadione, 1.25 mg; thiamine, 1.0 mg; riboflavin, 3.5 mg; d-pantothenic acid, 6.0 mg; vitamin B6, 1.5 mg; niacin, 27.5 mg; folic acid, 0.5 mg.

**Table 2:** Descriptive statistics on the effect of treatment on cecal colonization and categorized cecal loads^**^ of *Campylobacter jejuni* at 42 ds of age.

Cecal *C. jejuni* colonization	Treatment	
	Control	Added Fe- and iP-free
Mean^*^	3.78 × 10^8^	4.90 × 10^7^
Mean (log_10_CFU/g^1^)	8.58	7.69
SD of all measurements (10 birds per pen)	2.26	2.29
SE of all measurements (10 birds per pen)	0.226	0.229
SD of pen average (10 pens per treatment)	0.72	0.61
SE of pen average (10 pens per treatment)	0.228	0.193
Average CV per pen (individual bird variation)	45.0%	50%
Average CV of pens (between pen variation)	14.1%	13.1%
Median	2.26 × 10^5^	1.88 × 10^5^
Range	0.00 to 2.45 × 10^10^	0.00 to 2.97 × 10^9^
Rate (% positive birds out of 100 sampled)	88	84
Categories**		
Negative	12	16
<1 × 10^5^ CFU/g	20	24
1 × 10^5^ to 1 × 10^6^ CFU/g	38	40
1 × 10^6^ to 1 × 10^7^ CFU/g	17	11
1 × 10^7^ to 1 × 10^8^ CFU/g	7	7
>1 × 10^8^ CFU/g	6	2

1 Enumerated using culture-dependent methods. In brief: 10-fold serial dilutions of a 1 μL aliquot of cecal digesta in De Man–Rogosa–Sharpe broth were performed in a 96-well culture plate. The diluted samples were spread onto Campy-Cefex Agar plates (ThermoFisher Scientific) and incubated at 42 °C under microaerophilic conditions for 48 h prior to enumeration.

* Wilcoxon rank sum test on pen averages: *p*-value = 0.123.

** Chi-square test on categories (associationTest() function in R): *p*-value = 0.511.

Results from this small-scale experiment revealed that overall (0 to 42 d) growth performance—final body weight, body weight gain, mortality, and body weight-corrected feed conversion ratio—and livability did not differ significantly between the added Fe- and iP-free and control treatments (2,655 g/bird vs. 2,525 g/bird; 2,613 g/bird vs. 2,482 g/bird; 1.546 vs. 1.554; and 96.4% vs. 94.8%, respectively; *p*-value < 0.05 in all cases). Meanwhile, there was a 7.7-fold (87%) reduction in the average cecal *C. jejuni* load in the added Fe- and iP-free treatment relative to the control at 42 ds of age (4.90 × 10^7^ CFU/g vs. 3.78 × 10^8^ CFU/g, respectively; *p* = 0.12), as shown in Table 2. In addition, although the range in colonization loads was high (coefficient of variation was 45% among control-fed birds and 50% among birds fed the added Fe- and iP-free treatment), the upper end of the range was 1 log10 units lower in the added Fe- and iP-free treatment relative to the control 2.97 × 10^9^ vs. 2.45 × 10^10^, respectively.

## 5 Discussion

It is well known in the scientific community that *C. jejuni* counts in the broiler gut (especially in the ceca and feces) vary substantially between individual birds, even when birds are reared under controlled or standardized conditions (15). This often results in broad ranges or confidence intervals for group mean values, as observed in our study. Given this, it was considered that a 7.7-fold numerical reduction in mean cecal loads is of material interest. We also estimated that the average cecal *C. jejuni* load exhibited by the added Fe- and iP-free birds at 42 ds of age was below the level set by The European Food Safety Authority (EFSA) in 2011 as constituting an increased public health risk [>1,000 CFU/gram on broiler neck skin (16), equivalent to ~1 × 10^8^ CFU/g in the ceca according to the studies by (17, 18)]. This highlights the need for exhaustive additional research to evaluate the effect of dietary Fe, P, and phytase intervention on *Campylobacter* colonization in a larger-scale setting. The study design limited the possibility of separating the effects of the individual components of the intervention (Fe reduction, iP removal, and phytase supplementation) on bird responses. However, a reduced capacity of *C. jejuni* to colonize the cecum when dietary Fe and P were reduced is consistent with the existing (aforementioned) research, implicating Fe and P as nutritional virulence factors for the bacterium. The maintenance of growth performance in the added Fe- and iP-free-fed birds at a level not significantly different from that achieved by control-fed birds was likely afforded through the application of a higher dose of phytase supplementation (tiered by phase to reflect the higher nutrient requirements of younger birds).

The study we have presented provides suggestive evidence that small modifications to the diet composition to lower nutritional virulence factors, in combination with phytase supplementation, can help reduce *C. jejuni* colonization. These results suggest that targeted nutritional modifications can be potentially used as an additional approach to improve food safety, which has been overlooked until now. The authors recognize that individual variation among animals and a combined strategy approach limit the study findings. This variation is common to *Campylobacter* studies, as seen in previous published work, including Humphrey et al. (19). Hence, increasing replication power and conducting larger field-scale studies would help advance the ideas proposed herein and enhance our understanding.

## Data Availability

The raw data supporting the conclusions of this article will be made available by the authors, without undue reservation.

## References

[1] ECDC. Campylobacteriosis: Annual Epidemiological Report for 2022. Stockholm, Sweden: European Centre for Disease Prevention and Control (2024).

[2] Sahin O Morishita TY Zhang Q. Campylobacter colonization in poultry: sources of infection and modes of transmission. Anim Health Res Rev. (2002) 3:95–105. doi: 10.1079/ahrr200244, PMID: 12665109

[3] Zainol MFA Safiyanu MB Aziz SA Omar AR Chuang KP Mariatulqabtiah AR. Campylobacteriosis and control strategies against campylobacters in poultry farms. J Microbiol Biotechnol. (2024) 34:987–93. doi: 10.4014/jmb.2311.11045, PMID: 38719774 PMC11180925

[4] Oh E Andrews KJ Jeon B. Enhanced biofilm formation by ferrous and ferric iron through oxidative stress in *Campylobacter jejuni*. Front Microbiol. (2018) 9:1204. doi: 10.3389/fmicb.2018.01204, PMID: 29928267 PMC5998592

[5] Miller CE Williams PH Ketley JM. Pumping iron: mechanisms for iron uptake by Campylobacter. Microbiology. (2009) 155:3157–65. doi: 10.1099/mic.0.032425-0, PMID: 19696110

[6] Miller CE Rock JD Ridley KA Williams PH Ketley JM. Utilization of lactoferrin-bound and transferrin-bound iron by *Campylobacter jejuni*. J Bacteriol. (2008) 190:1900–11. doi: 10.1128/JB.01761-07, PMID: 18203832 PMC2258864

[7] Palyada K Threadgill D Stintzi A. Iron acquisition and regulation in *Campylobacter jejuni*. J Bacteriol. (2004) 186:4714–29. doi: 10.1128/JB.186.14.4714-4729.2004, PMID: 15231804 PMC438614

[8] Sinha R LeVeque RM Bowlin MQ Gray MJ DiRita VJ. Phosphate transporter PstSCAB of *Campylobacter jejuni* is a critical determinant of lactate-dependent growth and colonization in chickens. J Bacteriol. (2020) 202:e00716–9. doi: 10.1128/JB.00716-19 31932316 PMC7167465

[9] NRC. Nutrient requirements of poultry. 9th Revised ed. Washington, DC: The National Academies Press (1994).

[10] Menezes-Blackburn D Gabler S Greiner R. Performance of seven commercial phytases in an in vitro simulation of poultry digestive tract. J Agric Food Chem. (2015) 63:6142–9. doi: 10.1021/acs.jafc.5b01996, PMID: 26111064

[11] Selle PH Macelline SP Chrystal PV Liu SY. The contribution of phytate-degrading enzymes to chicken-meat production. Animals. (2023) 13:603. doi: 10.3390/ani13040603, PMID: 36830391 PMC9951704

[12] Marchal L Bello A Sobotik EB Archer G Dersjant-Li Y. A novel consensus bacterial 6-phytase variant completely replaced inorganic phosphate in broiler diets, maintaining growth performance and bone quality: data from two independent trials. Poult Sci. (2021) 100:100962. doi: 10.1016/j.psj.2020.12.059, PMID: 33652522 PMC7936205

[13] Philippi H Sommerfeld V Monteiro A Rodehutscord M Olukosi O. Impact of trace mineral source and phytase supplementation on prececal phytate degradation and mineral digestibility, bone mineralization, and tissue gene expression in broiler chickens. Biol Trace Elem Res. (2024) 202:5235–50. doi: 10.1007/s12011-024-04076 38329568 PMC11442561

[14] Dersjant-Li Y Kwakernaak C Bello A Marchal L. A novel consensus bacterial 6-phytase variant supplemented to an all-vegetable broiler diet totally replaced added trace minerals including zinc, iron, copper and manganese in two experiments. Poult Sci. (2025) 104:104610. doi: 10.1016/j.psk.2024.104610 39647354 PMC11667705

[15] Bahrndorff S Garcia AB Vigre H Nauta M Heegaard PMH Madsen M. Intestinal colonization of broiler chickens by *Campylobacter* spp. in an experimental infection study. Epidemiol Infect. (2014) 143:2381–9. doi: 10.1017/S0950268814003239, PMID: 25471550 PMC9150953

[16] EFSA. Scientific opinion on Campylobacter in broiler meat production: control options and performance objectives and/or targets at different stages of the food chain. EFSA J. (2011) 9:2105. doi: 10.2903/j.efsa.2011.2105 PMC13129689 42078736

[17] Brena MC. Effect of different poultry production methods on Campylobacter incidence and transmission in the broiler meat food chain, PhD thesis. Liverpool: University of Liverpool (2013).

[18] Emanowicz M Meade J Bolton D Golden O Gutierrez M Byrne W. The impact of key processing stages and flock variables on the prevalence and levels of *Campylobacter* on broiler carcasses. Food Microbiol. (2021) 95:103688. doi: 10.1016/j.fm.2020.103688 33397618

[19] Humphrey S Chaloner G Kemmett K Davidson N Williams N Kipar A. *Campylobacter jejuni* is not merely a commensal in commercial broiler chickens and affects bird welfare. MBio. (2014) 5:e01364–14. doi: 10.1128/mBio.01364-14 24987092 PMC4161246

[20] CVB (2021). CVB Feed Table 2021: Chemical composition and nutritional values of feedstuffs. Available online at: https://www.cvbdiervoeding.nl/bestand/10741/cvb-feed-table-2021.pdf.ashx (Accessed May 1, 2025)

[21] Beery JT Hugdahl MB Doyle MP. Colonization of gastrointestinal tracts of chicks by *Campylobacter jejuni*. Appl Environ Microbiol. (1998) 54:2365–70. doi: 10.1128/aem.54.10.2365-2370.1988 PMC204261 3060015

